# Fabrication of the Rapid Self-Assembly Hydrogels Loaded with Luteolin: Their Structural Characteristics and Protection Effect on Ulcerative Colitis

**DOI:** 10.3390/foods13071105

**Published:** 2024-04-04

**Authors:** Xin Bi, Han Peng, Hua Xiong, Lihua Xiao, Hua Zhang, Jiang Li, Yong Sun

**Affiliations:** 1 State Key Laboratory of Food Science and Resources, Nanchang University, Nanchang 330047, China; bixin@email.ncu.edu.cn (X.B.); huaxiong100@126.com (H.X.); ncuxiaolihua@163.com (L.X.); 2 Department of Food Science and Technology, University of California, Davis, 1 Shields Ave., Davis, CA 95616, USA; hanp@mun.ca; 3 College of Pharmacy, Jiangxi University of Traditional Chinese Medicine, Nanchang 330004, China; 20191022@jxutcm.edu.cn (H.Z.); viviface@yeah.net (J.L.)

**Keywords:** ulcerative colitis, luteolin, hydrogels, bioavailability, stimulation response

## Abstract

Luteolin (LUT) is a fat-soluble flavonoid known for its strong antioxidant and anti-inflammatory properties. Nonetheless, its use in the food industry has been limited due to its low water solubility and bioavailability. In this study, hyaluronic acid, histidine, and luteolin were self-assembled to construct tubular network hydrogels (HHL) to improve the gastrointestinal stability, bioavailability, and stimulation response of LUT. As anticipated, the HHL hydrogel’s mechanical strength and adhesion allow it to withstand the challenging gastrointestinal environment and effectively extend the duration of drug presence in the body. In vivo anti-inflammatory experiments showed that HHL hydrogel could successfully alleviate colitis induced by dextran sulfate sodium (DSS) in mice by reducing intestinal inflammation and restoring the integrity of the intestinal barrier. Moreover, HHL hydrogel also regulated the intestinal microorganisms of mice and promoted the production of short-chain fatty acids. The HHL hydrogel group demonstrated a notably superior treatment effect compared to the LUT group alone. The hydrogel delivery system is a novel method to improve the absorption of LUT, increasing its bioavailability and enhancing its pharmaceutical effects.

## 1. Introduction

Ulcerative colitis (UC) is a chronic inflammatory bowel disease that presents symptoms including diarrhea, bloody stools, a shortened colon, damaged mucosa, and ulcers within the colon. Thus, UC poses a serious health concern due to its high incidence, long duration, and high propensity to recur [1,2]. The development of ulcerative colitis is complex and involves a combination of factors, including the host’s environment, genetics, infections, and the intestinal microecosystem [3,4]. Nevertheless, medications for UC like 5-aminosalicylic acid, sulfasalazine, glucocorticoids, and immunosuppressants are generally costly and can be associated with significant side effects or a high likelihood of recurrence [5]. Therefore, a new treatment strategy with high efficacy but fewer side effects is desired.

Studies have shown that flavonoids present in vegetables, fruits, and herbs have excellent therapeutic effects on UC [6,7]. Luteolin, a flavonoid found in natural products like radicchio, oregano, and celery [8], has been shown to have multiple biological activities, including antioxidant, anti-inflammatory, antibacterial, pro-apoptotic, anti-tumor, liver protection, and other pharmacological effects [9,10,11]. Recent research has indicated that LUT may have positive effects on the gut microbiota and alleviate colonic injury and inflammation in UC rats [12,13]. Li et al. found that LUT could potentially inhibit experimental colitis by modulating the Nrf2 signaling pathway [14]. In this study, LUT was chosen as the primary focus of the research due to its exceptional pharmacological effects, as well as its extremely poor water solubility (1.93 × 10^−5^ mol/kg, 20 °C), poor absorption, rapid metabolism, and consequently low bioavailability following oral ingestion [15]. Therefore, increasing the solubility and bioavailability in vivo of LUT has been one of the hot spots in its research.

Hyaluronic acid (HA) is the main component of human connective cells/tissues such as interstitial cells, the vitreous body in the eye, and the synovial fluid in the joint [16]. HA is popular in the biomedical field due to its good biocompatibility, degradability, and immunogenicity [17]. More notably, HA binds explicitly to the overexpressed cluster of differentiation protein 44 (CD44) receptors on the surface of inflammatory cells [18]. The CD44 receptor is a glycoprotein that spans the cell membrane and is abundant on the surface of macrophages in inflamed colon tissue [19]. Therefore, using HA as a wall material promotes the active uptake of CD44-mediated macrophages through endocytosis [20,21]. Tie et al., designed the targeted treatment of mouse colitis with HA as a wall material loaded with procyanidin [22]. Another study noted that HA was grafted with histidine (His) to prepare nanogels for the targeted delivery of doxorubicin [23]. The preparation of hydrogels based on HA could promote the absorption and uptake of active ingredients at the inflammation site to achieve a better therapeutic effect [22]. Research has indicated that inflammation raises levels of acidic metabolites, resulting in a decrease in pH within the inflammatory environment [24]. Normal tissue has a pH of nearly 7.4, while inflammatory tissue has a pH of about 6.4 [25]. His, as a pH response fragment, has an imidazole ring with a pKa of around 6.0, which is close to the inflammatory tissue. Therefore, His is often used as a pH-responsive transport materials [26]. Zhang et al. developed pH-responsive intelligent nanovesicles based on poly(L-histidine) for precise drug delivery control [27]. Based on the unique pH and overexpression of CD44 receptors in inflammatory tissue, pH and CD44 receptor response hydrogels were designed.

Therefore, in this study, foodborne materials were used to create a stimulus-responsive luteolin-loaded hydrogel that could effectively treat the colitis in the mice model. Hydrogels were quickly self-assembled from HA, His, and LUT. The hydrogel’s morphology and structure were analyzed using cryo-scanning electron microscopy (SEM), X-ray diffraction (XRD), ultraviolet–visible (UV–Vis) spectroscopy, Fourier transform infrared spectroscopy (FTIR), and a rheometer. In addition, the hydrogel was stained with indocyanine green (ICG) instead of LUT, and the metabolism of the hydrogel in mice was observed. Meanwhile, the anti-inflammatory properties of the HHL hydrogel were evaluated in vivo in mice with DSS-induced ulcerative colitis. The efficacy of hydrogel therapy was evaluated by analyzing colon tissue samples, measuring inflammatory markers and tight junction proteins, studying the gut microbiota composition, and assessing the levels of short-chain fatty acids in mice with UC. The research may give some ideas for improving the bioavailability of LUT and UC treatments.

## 2. Materials and Methods

### 2.1. Materials

Hyaluronic acid was purchased from Shanghai Macklin Biochemical Co., Ltd. (Shanghai, China) (purity 97%). Histidine and Indocyanine green (ICG, purity, 75%) were obtained from Shanghai Aladdin Biochem Technology Co., Ltd. (Shanghai, China) (purity 99%). Luteolin was obtained from Shanghai Yuanye Biological Technology Co., Ltd. (Shanghai, China) (purity, 96%). C57BL/6 male mice (6 weeks) were purchased from the Hunan SJA Animal Laboratory (Changsha, China). Dextran sulfate sodium salt (DSS, molecular weight 36,000–50,000 Dalton, colitis grade) was purchased from MP Biomedicals (Shanghai, China) Co., Ltd. Interleukin-1β (IL-1β), interleukin-6 (IL-6), and mice tumor necrosis factor-α (TNF-α) ELISA kits were obtained from Meimian Industrial Co., Ltd. (Yancheng, China).

### 2.2. Preparation of HHL Hydrogel

HA-His was synthesized according to the previous literature [23,28]. Briefly, 400 mg of HA was dissolved in 10 mL of ultrapure water. The solution was stirred for 4 h until it was completely dissolved. His (232 mg) was added and reacted for 24 h. Subsequently, 20 mg of LUT was added to the HA-His solution, stirred, and heated to 95 °C for 20 min. Subsequently, it was cooled down to room temperature, resulting in the formation of a stable HHL hydrogel.

### 2.3. Characterization of HHL Hydrogel

The hydrogel’s morphology was examined through scanning electron microscopy (SEM, JSM 6701F, XFlash6, Tokyo, Japan). Simultaneously, the hydrogel was freeze-dried and ground into a powder. It was then flattened and scanned with an X-ray diffractometer. (ESCALAB250Xi, Thermo Fisher Scientific, Waltham, MA, USA) [28]. The FTIR spectra of the hydrogel were analyzed utilizing an FTIR spectrometer (Nicolet 5700, PerkinElmer, Waltham, MA, USA) with the KBr tablet method [29], scanning within the range of 4000 to 400 cm^−1^. The UV spectra of appropriate concentrations of HA, His, LUT, and HHL hydrogels were measured with a spectrophotometer (TU-1900, PERSEE, Beijing, China) employing a cuvette with a 0.01 mm optical path at room temperature [30].

### 2.4. Rheology Analysis

The TA instrument was used to analyze the rheological characteristics of hydrogels. (DHR-203052207, WATER, Holly Hollow, TX, USA). The diameter of the plate fixture was 40 mm, and the gap between the plates was 1 mm. The testing frequency ranged from 1 to 100 Hz with a consistent 1% strain, and the testing temperature was 25 °C. The dynamic strain scan measurements were conducted by cycling between 1% and 300% strain in three consecutive cycles lasting 600 s [31]. 

### 2.5. Stability of HHL Hydrogel

A 20 mL pH 2, pH 6.4, or 7.4 PBS buffer was injected into 1 mL of HHL hydrogel and incubated at 37 °C. The degradation of hydrogel was observed in different time periods [32].

### 2.6. Biodistribution of Hydrogel in Mice

Hydrogels loaded with ICG were prepared by substituting LUT with ICG. Mice (C57BL/6 male, 6–8 weeks) were administered ICG (ICG group) or ICG-loaded hydrogel (HH−ICG group) at a dose of HA equivalent of 100 mg/kg (*n* = 3 in each group) [30]. The hydrogel’s distribution was evaluated at specific time intervals (3, 6, 9, and 12 h) using an in vivo imaging system (IVIS, AniView 600, Biolight Biotechnology, Guangzhou, China). Then, the gastrointestinal tract of mice was separated, and the distribution of hydrogel in the gastrointestinal tract was observed via the IVIS system. Approval for the animal experiments was granted by the Experimental Animal Science and Technology Center of Jiangxi University of Chinese Medicine (TEMPOR20230128, 8 November 2023). All animal housing and experiments were conducted in strict accordance with the institutional guidelines for the care and use of laboratory animals.

### 2.7. Intervention of HHL Hydrogel on DSS-Induced Colitis in Mice

After a week of day/night cycle adaptations, the mice were divided into 4 groups with 12 mice in each group: the control group (CON group: receiving untreated drinking water); the model group with DSS (MOD group: 3% DSS); the LUT and DSS-treated group (LUT group: 3% DSS+LUT at 5 mg/kg); and the HHL and DSS-treated group (HHL group: 3% DSS+HHL hydrogel with LUT equivalent of 5 mg/kg). Between the 15th and the 21st day, the mice in the group treated with DSS were given water that contained 3% DSS, resulting in the development of colitis. From the 8th day to the 21st day, the LUT and HHL groups received a dosage equivalent to 5 mg/kg of LUT. Throughout the modeling period, the mice’s body weight, rectal bleeding, fecal hardness, and behavior were monitored and documented daily for analysis of the disease activity index (DAI) [14]. The animal experiments were approved by the Experimental Animal Science and Technology Center of the Jiangxi University of Chinese Medicine (JZLLSC20220492, 29 March 2022). All animal housing and experiments were conducted in strict accordance with the institutional guidelines for the care and use of laboratory animals.

Following the treatments, the mice were euthanized after a 12 h fasting period, and their eyeballs were extracted to collect blood. The serum, viscera, colon, and caecum contents were collected and stored in a −80 refrigerator. The mice colon was divided into 4 parts for H&E staining, RT-PCR, and inflammation-related indicator analysis.

### 2.8. Histological and Immunohistochemical Analyses of Colon Tissue

The colon tissue was rinsed multiple times with a saline solution, then treated with 4% paraformaldehyde for fixation. Afterward, the samples underwent ethanol dehydration, were encased in paraffin, sectioned, and finally stained with Hematoxylin and Eosin (H&E). Inflammatory damage to the colon was examined using an optical microscope. A portion of colon tissue was examined for zonula occludens-1 (ZO-1) immune fluorescence using a Ti-S fluorescent inverted microscope manufactured by Nikon Corporation in Tokyo, Japan [33,34].

### 2.9. In Vivo Anti-Inflammatory Effects Study

The colon tissues were weighed, mixed with sterile PBS at a ratio of 1:9, with a pH of 7.4, and then centrifuged to collect the supernatant. The ELISA kits were utilized to quantify the amounts of TNF-α, IL-1β, and IL-in the supernatant.

### 2.10. Real-Time Quantitative PCR Analysis (RT-qPCR)

The colon tissues were used to extract total RNA using an RNA extraction kit from Beyotime (Shanghai, China), followed by determining the RNA concentration. The extracted RNA was then reverse-transcribed into cDNA using the Hifair V one-step RT-gDNA digestion SuperMix for qPCR kit (Shanghai, China). The reversed-transcribed cDNA was treated with TB Green Premix Ex Taq II (TliRNaseH Plus) from TaKaRa, (Kusatsu, Shiga, Japan), and then quantified using RT-PCR equipment from Biorad (Redmond, WA, USA). The reference gene used was β-actin, and the expression levels of the relevant genes were determined using the 2^−ΔΔct^ method. The primer sequences for ZO-1, Occludin, and Claudin-1 can be found in Table S2 [35,36].

### 2.11. Analysis of the Gut Microbiota

Following the sacrifice of the mice, the cecal contents were gathered, and the intestinal microbiota was studied using 16S rDNA amplicon sequencing technology. Sample handling included procedures such as DNA extraction, PCR amplification, quantification of fluorescence, construction of Miseq libraries, and Miseq sequencing. These tasks were carried out at Shanghai Majorbio Biopharm Technology Co., Ltd., located in Shanghai, China.

### 2.12. Determination of Content of SCFAs

The assessment of short-chain fatty acid (SCFA) levels was conducted using a method that had been previously reported, with minor adjustments [37]. Fecal samples (50 mg) were combined with deionized water (0.35 mL) and 10% sulfuric acid (17.5 μL). Following a 20 min incubation period, the supernatant was retrieved by centrifuging the mixture at 10,000× *g* for 15 min at 4 °C. Then, the above operation was repeated once. The double supernatant was passed through a 0.22 μm filter membrane. The Agilent 7890B gas chromatography (Agilent, Santa Clara, CA, USA) system was used to detect the content of SCFAs on the Agilent 19091F-413 column [38]. 

### 2.13. Statistical Analysis

All the results were expressed as the mean ± SD. The study used the one-way analysis of variance (ANOVA) and Duncan’s multiple-range test to examine the significant distinctions between groups, with data analysis conducted through SPSS 22.0 software from Chicago, USA. A *p*-value of < 0.05 denotes a statistically significant difference.

## 3. Results and Discussion

### 3.1. Characterization of the Hydrogel

**Figure 1:**
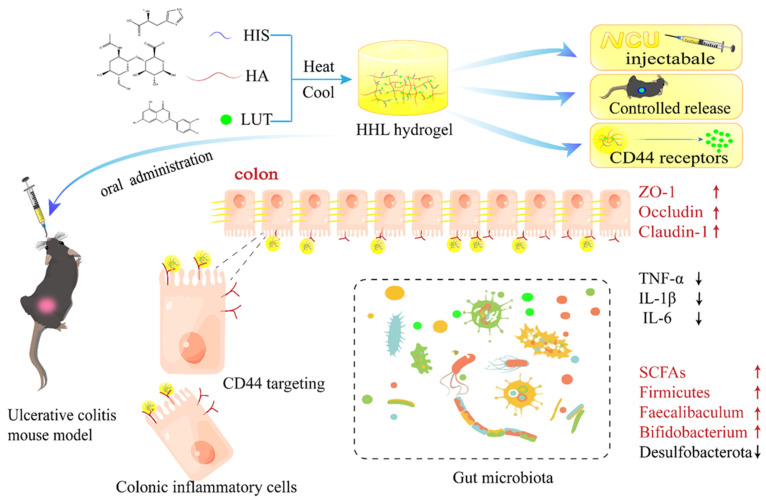
Schematic diagram of HHL hydrogel preparation and application in relieving a DSS-induced colitis model.

**Figure 2:**
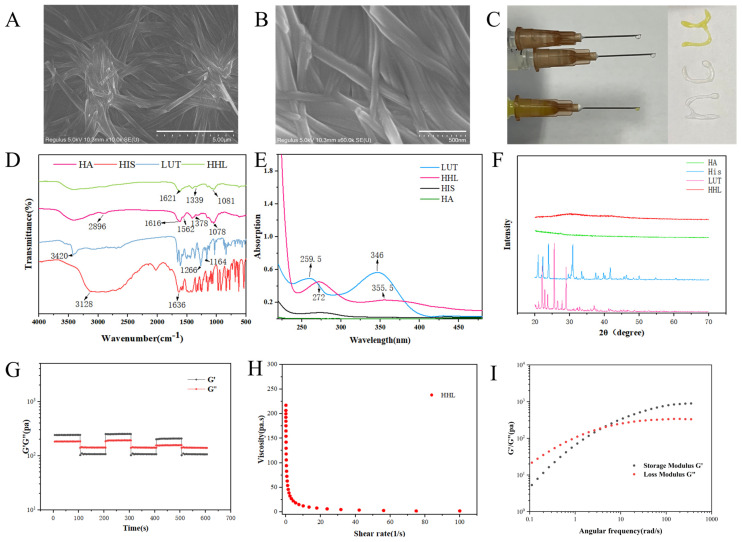
Characterization of HHL hydrogel. (**A**,**B**) The SEM result of the HHL hydrogel, scale bar: 5 μm and 500 nm. (**C**) Photographs demonstrating the injectability and filling capacity of H, HH, and HHL hydrogels. (**D**) FTIR spectra; (**E**) UV spectra; (**F**) X-ray diffraction patterns of HA, HIS, LUT, and HHL. (**G**) The thixotropic recovery properties of HHL hydrogels. (**H**) Shear viscosity curve of HHL hydrogel. (**I**) Frequency sweep curve of constant strain (1%).

As shown in Figure 1, HA, His, and LUT were mixed, heated, and cooled to obtain stable yellow hydrogels. The SEM was utilized to analyze the microscopic structure of HHL hydrogel, and it could be clearly displayed that the hydrogel consisted of multiple intertwined tubular network structures (Figure 2A,B). The network structure of hydrogels could trap a large amount of water in them and lose their fluidity [39]. Therefore, hydrogels have solid-like properties with excellent mechanical strength and adhesion, which is conducive to the use of hydrogels for the delivery of active ingredients in vivo. As shown in Figure 2C, the hydrogel could be smoothly pushed out of the syringe and take on a specific shape. The injectability of the hydrogel enables efficient administration of LUT, making it possible for drug delivery.

The self-assembly mechanism of HHL hydrogels was investigated by FT-IR, UV–Vis, and XRD technologies. The characteristic absorption peaks of HA show the following vibrations: the stretching vibration of −OH at 3402 cm^−1^, the in-plane bending vibration of O−H at 1378 cm^−1^, the stretching vibration of C=O at 1616 cm^−1^, and the bending vibration of N−H at 1562 cm^−1^ (Figure 2D). After the formation of the HHL hydrogel, the in-plane bending vibration of O−H changed from 1378 cm^−1^ to 1339 cm^−1^, and the bending vibration of N−H at 1562 cm^−1^ almost disappeared. This change is likely attributed to the development of hydrogen bonds in the hydrogel’s self-assembly process, leading to a redshift in wavelength [40,41]. Moreover, the characteristic absorption peak of LUT almost disappeared after the formation of hydrogel, indicating that LUT was successfully encapsulated. Subsequently, the change in UV-Vis spectra of different components was observed. The characteristic absorption peaks of LUT at 259.5 nm and 346 nm were shifted to 272 nm and 355.5 nm in HHL hydrogel, indicating the existence of π–π interaction after hydrogel formation (Figure 2E) [42]. At the same time, the characteristic absorption peak of LUT at 346 nm nearly disappeared, which once again proved that LUT was successfully packaged. Subsequently, the crystalline structure and amorphous structure of HA, His, LUT, and HHL were studied by XRD. It has been suggested in many studies that the crystalline structure of the materials disappears after encapsulation [43,44]. His and LUT were found in the crystalline structure, while HA was in an amorphous state. After forming the HHL hydrogel, there was a decrease in the intensity of the diffraction peak of His (Figure 2F). This indicated that the crystalline structure of His was partially disrupted during the process of hydrogel formation. Notably, the XRD diffraction pattern of LUT completely disappeared after treatment, which may be related to the materials’ molar ratio (HIS: LUT~21.4:1) in the HHL hydrogel. The molecular arrangement of LUT is covered or encapsulated. This result of XRD is consistent with those of UV–Vis and FT-IR. Wang et al., reported that the fibrous hydrogels were rapidly formed after adding a few resveratrols to the gallic acid aqueous solution after heating. These phenolics interact with each other via hydrogen bounds and π–π interactions [29]. Our research has also validated that the hydrogels form through hydrogen bonding and π–π stacking interactions among the three substances, without the involvement of covalent bonds.

### 3.2. Rheological Analysis

The researchers utilized oscillatory shear rheology to examine the thixotropic recovery characteristics of the HHL hydrogels. The low strain (1%) and high strain (300%) modes were applied alternately for three consecutive cycles lasting 600 s. The stability of the HHL hydrogel was indicated when the G’ value exceeded the G” value under a 1% strain. However, as the strain reaches 300%, the G’ value becomes lower than the G”, signaling a transition in the hydrogel’s state from gel to sol [45] (Figure 2G). The mechanical properties of HHL hydrogels recovered after the strain decreased (from 300% to 1%), suggesting that HHL hydrogels have good thixotropic recovery properties [45]. The G’ decrease occurred in the third cycle of the high- and low-strain transition. The reason for this phenomenon may be the partial disruption of hydrogel interactions under high strains. Research was conducted on the shear-thinning characteristic of hydrogel. The findings revealed that with higher shear rates, the hydrogel’s viscosity decreased, demonstrating shear-thinning behavior. (Figure 2H). The thixotropic recovery properties and mechanical strength of hydrogels can potentiate their application as drug delivery systems [46,47]. The frequency scanning curve of constant strain is shown in Figure 2I. The modulus of HHL hydrogel had an obvious frequency dependence, and G’ and G” gradually increased with the increase in angular velocity. When the angular velocity was small, G’ was smaller than G”, and the hydrogel was in a flowable sol state. When the angular velocity increased, G’ was greater than G”, indicating that the hydrogel exhibited solid-like properties. The intersection of G’ and G” was called the gel point. This is typical of the rheological properties of dynamically crosslinked network hydrogels [48]. The reason for this phenomenon may be that HHL hydrogels have weak intermolecular interaction forces in the low-frequency environment, and with the increase in frequency, the intermolecular rearrangements are rapid, the intermolecular interactions become tighter, and the G’ of the hydrogel thus becomes larger [49].

### 3.3. Stability Evaluation and Biodistribution of HHL Hydrogel In Vivo

To examine the pH stability of hydrogels, various PBS buffers with different pH levels were introduced to the hydrogels. As depicted in Figure S2, the degradation of the hydrogel in the pH 2 buffer was significantly greater compared to the degradation in the pH 6.4 and 7.4 buffers. HHL hydrogel was partially degraded after being stored in pH 2 buffer for 3 h, and it was completely degraded after 6 h. The degradation rate in the buffer of pH 6.4 was faster than that of 7.4 buffers. However, hydrogels were relatively stable in 7.4 buffers, and some hydrogels were still present after 12 h. The findings showed that the hydrogel remained stable in neutral conditions but that it could be easily degraded in an acidic environment.

**Figure 3:**
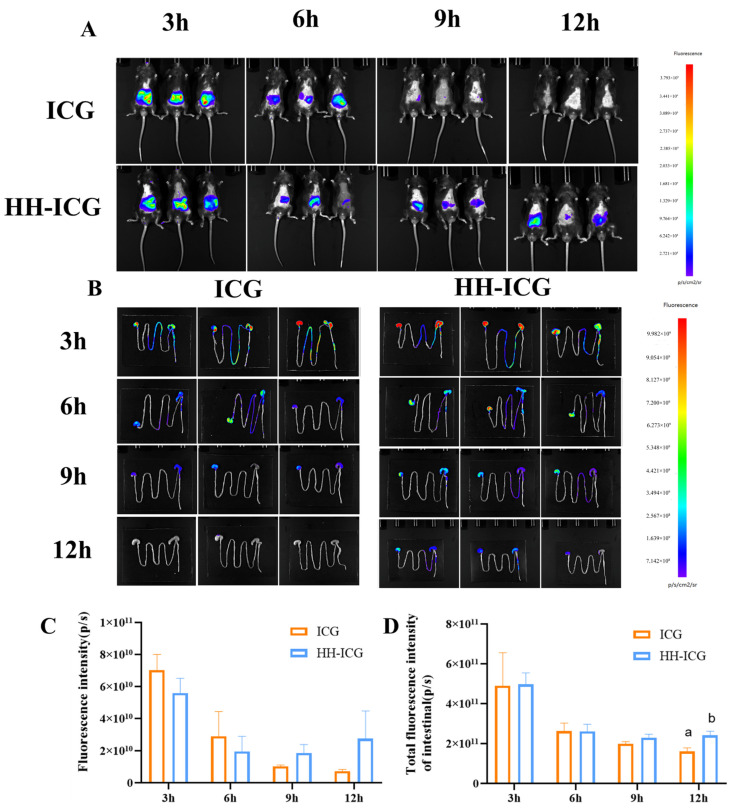
Degradation of HHL hydrogel and biodistribution of ICG/HH-ICG in mice: (**A**) Distribution of fluorescence in mice at various time points. (**B**) Fluorescence imaging of gastrointestinal tract in mice at different time periods. (**C**) The quantification of fluorescence intensity in mice at different time point. (**D**) Fluorescence quantification of the gastrointestinal system. The values are mean ± SD (*n* = 3). Different letters in each column indicate significant differences between groups at the same time point (*p* < 0.05).

To further observe the stability of HHL hydrogel in the gastrointestinal tract, ICG was used as a fluorescence tracer instead of LUT, and the in vivo imaging system was utilized to observe the distribution of the hydrogel in mice. The whole-body fluorescence intensity was stronger in mice from the ICG group compared to the HH-ICG group after either 3 h or 6 h of oral administration. The fluorescence of the ICG group was very weak after 9 h, and it nearly disappeared after 12 h (Figure 3A,B). The fluorescence intensity in the ICG group (7.24 × 10^9^ ± 1.09 × 10^9^ p/s) decreased compared to the HH-ICG group (2.74 × 10^10^ ± 1.734 × 10^10^ p/s) after 12 h. In addition, in this experiment, the gastrointestinal systems of mice were stripped to further investigate the distribution of ICG in the gastrointestinal tract. In line with the findings of the whole body of mice, the intestinal fluorescence intensity of mice in the hydrogel group was (2.40 × 10^10^ ± 2.155 × 10^10^ p/s) markedly stronger than that in the ICG group (1.60 × 10^10^ ± 1.819 × 10^10^ p/s) after 12 h of oral administration (*p* < 0.05) (Figure 3C,D). 

The results from the pH stability experiment were in line with those of the animal experiment, and some hydrogels did not degrade after 3 h in a strong acid environment but were stable in a neutral environment of the intestine. These phenomena may be due to the adhesion properties of hydrogels, which can delay the metabolism of ICG, indicating that the hydrogel could prolong the amount of time the drug remains in mice and improve the bioavailability of the drug. Zhang et al., also developed a drug delivery system using a hyaluronic acid/gelatin hydrogel to extend the presence of curcumin in the digestive system [50]. Another research study found that a hydrogel created from a combination of gallic acid and sodium alginate exhibited impressive mucoadhesive properties and was able to effectively remain in the intestinal tract for an extended period of time [51]. Therefore, due to their mechanical strength and adhesive properties, hydrogels may help establish a gastrointestinal sustained release delivery system for certain pharmaceutical ingredients.

### 3.4. Effect of Hydrogel on DSS-Induced Colitis

**Figure 4:**
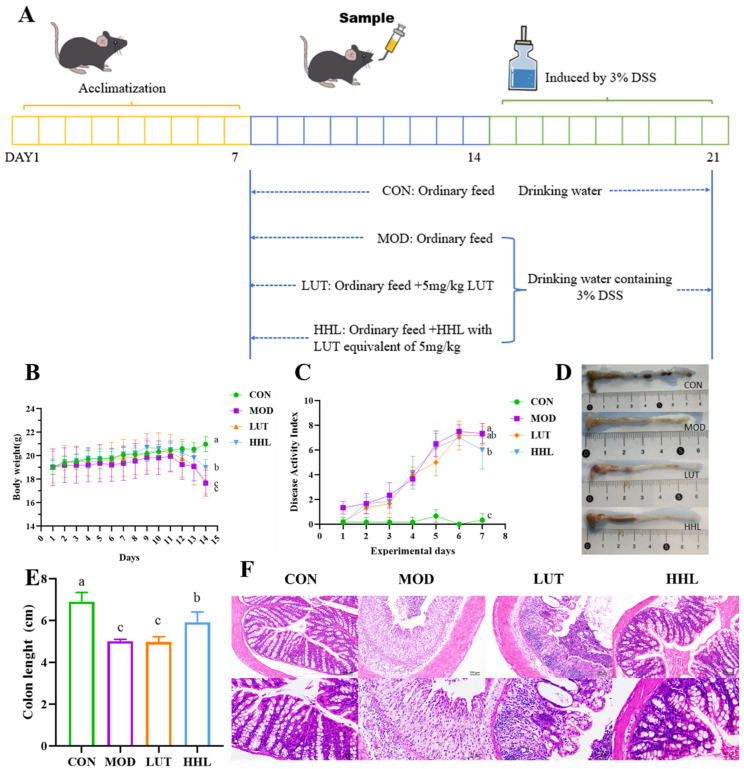
Anti-inflammatory activity of hydrogel in vivo. (**A**) Diagram of colon inflammation experiment in vivo. (**B**) Daily weight change. (**C**) DAI scores. (**D**) Colons of different groups of mice. (**E**) Colon length of different groups of mice (a–c). (**F**) H&E staining results. The values are mean ± SD (*n* = 6).

Subsequently, this study further investigated whether HHL hydrogel could improve the intervention effect of LUT on colitis in mice. Starting on day 18, the mice in the DSS treatment group started to exhibit weight loss. On the last day, the body weight of mice in the HHL hydrogel group was significantly higher than that in the MOD group, but no significant effect was seen in the LUT group (*p* < 0.05) (Figure 4B). Therefore, the HHL hydrogels successfully reduce the weight loss induced by DSS in mice.

Throughout the modeling phase, the body weight, vitality, fur condition, posture, and rectal bleeding of the mice were monitored on a daily basis (Table S1). The DAI score of mice in the HHL group (6.99 ± 1.549) showed a significant decrease compared to the MOD group (7.33 ± 0.816) (*p* < 0.05), but the pathological state still existed (Figure 4C). The DAI score of mice in the LUT group (7.17 ± 0.753) was similar to that in the MOD group. The results indicated that HHL hydrogel could effectively alleviate the blood in stool and diarrhea caused by DSS in mice.

Following treatment with DSS, the length of the colons in mice notably decreased, and HHL hydrogel could effectively alleviate the colon shortening induced by DSS (Figure 4D,E). Compared with the colon length (5.01 ± 0.09 cm and 5.00 ± 0.25727 cm) of the MOD and LUT groups, the colon length of the HHL group was 5.92 ± 0.49 cm, showing a significant difference (*p* < 0.05).

The colon tissue sections were examined using H&E staining to assess the damage. In the CON group, the colon sections of mice showed an intact structure with well-organized crypts and glands. Obvious inflammatory cell invasion, destruction of the crypt structure, and reduction in goblet cells were observed in the colon sections of the MOD and LUT groups (Figure 4F). It is important to mention that the colon structure of the HHL group was comparable to that of the CON group, suggesting that HHL hydrogel displayed impressive anti-inflammatory properties and was effective in alleviating colitis in mice. 

**Figure 5:**
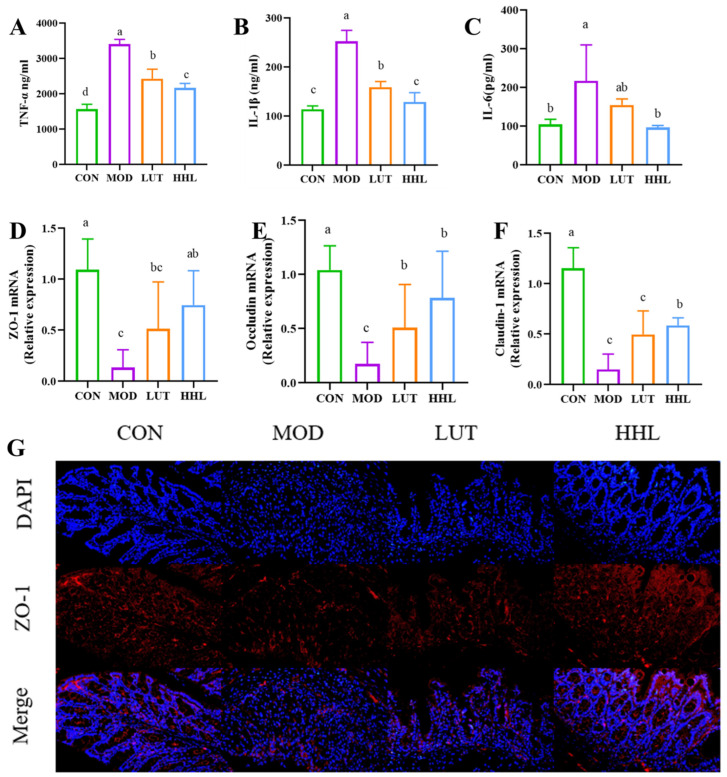
Colonic inflammatory indicator level. (**A**–**C**) Expression of inflammatory factors in colonic sites in different treatment groups (a–c), (**D**–**F**) The mRNA expression levels of ZO-1, Occludin and Claudin-1 in colon (a–c). (**G**) Fluorescence image of ZO-1 at 200 times (scale bars = 100 μm) (*p* < 0.05).

The extent of colon inflammation was evaluated through the measurement of TNF-α, IL-1β, and IL-6. The levels of these three factors in the MOD group were markedly higher than those in the CON group, indicating that DSS could cause these inflammatory factors to accumulate in colon tissue. The HHL group showed significantly lower levels of TNF-α, IL-1β, and IL-6 compared to the MOD group (*p* < 0.05) and similar to those in the CON group. While LUT could reduce the release of inflammatory cytokines, the impact of HHL hydrogel is even more substantial (Figure 5A–C). 

The HHL hydrogel exhibited outstanding therapeutic effectiveness in treating DSS−induced colon damage. Mice treated with HHL hydrogel showed a significant reduction in weight loss, reduced bleeding, and reduced colon shortening. In addition, we conclude that HHL hydrogel could protect the structure and reduce inflammation of the colon. It is worth mentioning that the HHL hydrogel showed a better intervention effect than LUT alone against DSS-induced colitis. Multiple research studies have revealed that the impressive anti-inflammatory characteristics of LUT are impeded by its inadequate water solubility and restricted bioavailability [8,15]. We hypothesized that HHL hydrogels could enhance the therapeutic effect of LUT. The sustained delivery of the drug to the colon site may be facilitated by the mechanical strength and adhesion of the hydrogel in the intestine. In addition, HA could bind to the over-expressed CD44 receptor on the surface of inflammatory cells, further prolonging the residence time of hydrogels at the site of inflammation and ultimately achieving the precise release of LUT. These results demonstrate the utility of constructing stimulus-responsive hydrogels.

### 3.5. Effect of Tight Junction Protein

Proteins like ZO-1, Occludin, and Claudin-1 form tight junctions to link intestinal epithelial cells together and maintain the integrity of the intestinal barrier. It was reported that reduced expression of tight junction protein is positively correlated with intestinal injury [52]. The gene expression levels of three proteins were measured by PCR. As a result, the contents of these tight junction proteins were markedly reduced after DSS treatment. Surprisingly, following administration of HHL hydrogel, the mRNA expression of three proteins was significantly increased compared with the MOD group. In addition, the mRNA expression of Claudin-1 protein was significantly increased in the HHL group compared to the LUT group (*p* < 0.05) (Figure 5D,F). In order to directly assess the protein expression level, immunofluorescence analysis was conducted to examine the expression of ZO-1 in colon tissue. The expression of ZO-1 in the MOD and LUT groups was significantly lower than that in the CON and HHL groups (Figure 5G). Significantly, the level of ZO-1 expression in the HHL group was similar to that in the CON group. The results showed that using HHL hydrogel is more effective in preserving the integrity of the intestinal mucosal barrier compared to using LUT alone. Maintaining the integrity of the intestinal barrier is crucial for preventing the passage of harmful substances [53]. Injuries to the intestinal barrier have been linked to various illnesses such as intestinal conditions (IBD, irritable bowel syndrome, and colon cancer) and systemic diseases (such as non-alcoholic fatty liver disease, Parkinson’s disease, and depression) [54]. In combination with H&E staining results, the HHL group was shown to reduce DSS-induced goblet cell loss, mucosal injury severity, and tight junction protein expression. The results indicate that HHL hydrogel could effectively relieve intestinal barrier damage caused by DSS. 

### 3.6. Effect of Gut Microbiota

**Figure 6:**
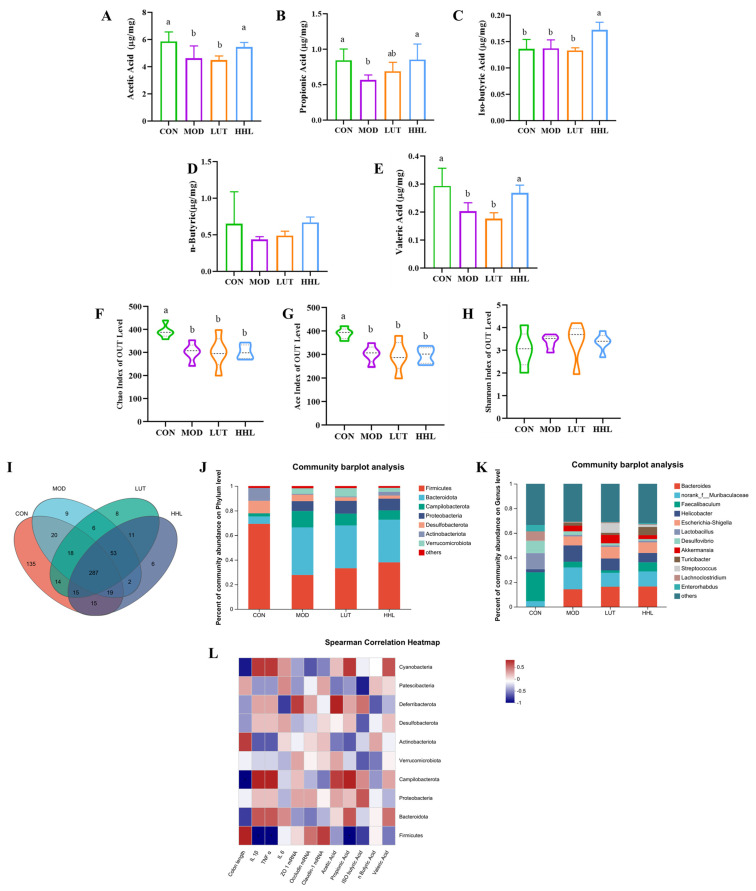
Effects of HHL hydrogel on SCFAs and diversity and species composition of gut microbiota in DSS mice. The levels of SCFA metabolites in fecal (**A**) acetic acid, (**B**) propionic acid, (**C**) iso-butyric acid, (**D**) n-butyric acid, and (**E**) valeric acid. Gut microbiota, (**F**) Chao index, (**G**) Ace index, and (**H**) Shannon index of operational taxonomic unit (OTU) level analysis in different treatment groups. (**I**) Venn diagram of shared and unique bacteria at the OTU level. (**J**) The relative abundance histogram of gut microbiota at the phylum level in different treatment groups (a, b). (**K**) The relative abundance histogram of gut microbiota at the genus level. (**L**) Correlation between phylum-level microflora and intestinal inflammatory indices of mice in the HHL group through Spearman correlation analysis.

Colitis can cause an imbalance of the gut microbiota [55]. And SCFAs are the end products of beneficial intestinal fermentation, which is closely related to gut microbiota [56]. Studies have shown that SCFAs are crucial for preserving the morphology and structure of colon epithelial cells, and they could be used to effectively protect against intestinal diseases [57]. In this study, the content of five short-chain fatty acids was determined by gas chromatography. As shown in Figure 6A–F, the contents of SCFAs, including acetic acid, propionic acid, iso-butyric acid, n-butyric acid, and valeric acid, were all low in the MOD group, and there was no obvious improvement after LUT treatment. It is important to highlight that following HHL hydrogel treatment, the contents of acetic acid, propionic acid, and valeric acid were extremely close to those of the CON group. In addition, the HHL hydrogel could significantly increase the secretion of iso-butyric acid (*p* < 0.05). The results indicated that HHL hydrogel promoted the production of SCFAs.

The creation of SCFAs in the intestine is largely controlled by microbial metabolism. It is probable that the HHL hydrogel increased SCFA levels by influencing the gut microbiota. In addition, the gut microbiota composition of mice in each group was analyzed by 16S rDNA amplification sequencing of cecal contents. Alpha diversity index (Chao index, Ace index and Shannon index) was used to analyze the richness and diversity of microbial communities in each group. DSS treatment decreased the microbial community richness, while the oral administration of LUT and HHL hydrogels had no remarkable effect on community richness (Figure 6F,G). Compared with the CON group, the MOD group, LUT group, and HHL group had no significant changes in community diversity (Figure 6H). The Venn diagram illustrated that there were 287 operational taxonomic units (OTUs) shared by every group. The number of unique OTUs in the CON, MOD, LUT, and HHL groups was 135, 9, 8, and 6, respectively (Figure 6I). There was no significant difference in the richness and diversity of the gut microbiome among the different treatment groups, which aligns with the findings reported by Feng et al. [58]. β diversity is an indicator to assess the similarity of intestinal microflora between different treatment groups. PCA and PCoA were used to examine the composition of intestinal microflora among different groups. As shown in S4A and S4B, the community composition of the HHL group was closest to that of the CON group, and there was some overlap, indicating that the community composition was similar.

The community Bar diagram was utilized to depict the distribution of dominant species in samples from each group at different levels for further evaluation. According to Figure 6J, treatment with DSS may lead to alterations in the composition of the intestinal microbiota. As could be obtained from the phylum level, DSS caused a decrease in the abundance of *Firmicutes*, while the HHL hydrogel intervention increased the abundance of *Firmicutes*. The species composition at the genus level was further analyzed. The DSS treatment led to a higher presence of harmful bacteria, such as *Helicobacter*, which negatively relieved colon inflammation (Figure 6K) [59]. However, HHL hydrogel intervention reduced the abundance of Helicobacter. In comparison to the CON group, the MOD and LUT groups showed a decrease in the abundance of *Facecalibaculum*, while the HHL group exhibited an increase. Research has indicated that UC patients have low levels of *Faecalibaculum*. Additionally, metabolites from *Faecalibaculum* have been found to boost the presence of SCFAs, with a particular focus on butyric acid. Butyric acid aids in repairing the intestinal mucosa and hinders the release of proinflammatory cytokines from neutrophils in IBD [60,61,62,63]. Consistent with the SCFA levels, the butyric acid levels in the HHL group were higher than those in other groups. Moreover, LUT treatment alone did not increase the abundance of beneficial bacteria in mice. The reason for this phenomenon could be a result of the low solubility of LUT in water, which leads to rapid metabolism in mice, resulting in the failure of LUT to fully exert its influence on the gut microbiota.

LEfSe (linear discriminant analysis effect size) was used to further examine bacteria, showing significant statistical differences among the groups. The LDA value distribution histogram and evolutionary branching diagram were used to display. The results, as shown in Figure S4C, reveal that the MOD group showed enrichment of the phylum *Desulfobacterota* compared with the HHL group. *Desulfobacterota*, a pathogenic bacterium, can promote intestinal inflammation and epithelial permeability [64]. It should be noted that *Actinobacteriota* was considered to be an abundant bacterial phylum in the HHL group. Moreover, *Bifidobacterium*, which belongs to *Actinobacteriota*, is also the dominant species after HHL hydrogel treatment. There are reports suggesting that *Bifidobacterium* may improve the barrier function of mice with necrotizing enteritis [65]. These findings suggest that HHL hydrogel is a positive regulator of the gut microbiota.

Spearman correlation analysis was used to investigate the relationship between different bacterial flora in the HHL group and colitis indicators such as colon length, inflammatory factors, tight-junction protein, short-chain fatty acids, etc. There was a positive relationship between the abundance of *Firmicutes* and colon length as well as Claudin-1 mRNA content, while a negative correlation was observed with IL-1β, TNF-α, and propionic acid content (Figure 6L). This outcome aligns with the one documented by Wu et al. [59]. The HHL hydrogel showed a tendency to recover the abundance of Firmicutes, indicating that the intestinal barrier damage and inflammation were less severe in the HHL hydrogel mice. Nevertheless, there was a negative correlation between the prevalence of *Cyanobacteria* and *Campilobacterota* and colon length, while a positive correlation was observed with IL-1β and TNF-α. Research has indicated that *Cyanobacteria* have the potential to release a toxin known as microcystin-leucine, which could exacerbate and prolong the severity of UC [66]. Therefore, the HHL hydrogel has the potential to lower levels of inflammatory factors, demonstrate anti-inflammatory effects, and decrease colon shortening by influencing the populations of *Firmicutes*, *Cyanobacteria*, and *Campilobacterota*. 

Based on the gut microbiota data, after inducing colitis in mice, the equilibrium of the intestinal microbiota was disturbed. In this study, the effect of LUT on SAFCs and gut microbiota was not obvious, which may be due to the low dose of the LUT group, and the poor water solubility of LUT led to rapid metabolism in mice, resulting in the failure of LUT to fully exert its pharmacological activity. The hydrogel system could effectively delay the metabolism of the drug so that the drug could continue to exert its pharmacological effect at the colonic site. Therefore, in the hydrogel encapsulation, LUT could have a better therapeutic effect. It is important to mention that the presence of helpful bacteria, such as *Firmicutes*, *Faecalibaculum*, and *Bifidobacterium*, rose following the HHL hydrogel treatment. Under the action of these beneficial bacteria, the intestinal inflammatory response is reduced, the intestinal barrier is repaired, and more SCFAs are produced. As a result, the HHL hydrogel effectively regulates the gut microbiota.

## 4. Conclusions

In summary, the HHL hydrogel could be fabricated through hydrogen bonding, conjugation, and other intermolecular interactions. The mechanical strength and CD44 receptor response of the hydrogel prolonged the retention time of LUT in mice and subsequently acted more effectively as a therapeutic agent against the disease. Moreover, the HHL hydrogel has the ability to mitigate DSS-induced colitis in mice through mechanisms such as decreasing inflammatory factors, boosting levels of intestinal tight junction proteins, generating SCFAs, and balancing the gut microbiota. This study provides a simple method to improve the bioavailability of LUT and highlights the effectiveness of hydrogels in developing controlled-release delivery systems for bioactive compounds aimed at treating UC. However, more targeting mechanisms of hydrogels in the colitis model still need to be further verified.

## Data Availability

The data presented in this study are available on request from the corresponding author.

## Supplementary Materials

The following supporting information can be downloaded at https://www.mdpi.com/article/10.3390/foods13071105/s1, Figure S1: The structural information of HA, His and LUT; Figure S2: The pH stability of HHL hydrogels; Figure S3: The food intake during animal experiments; Figure S4: Gut microbiota composition and differential species analysis of DSS induced colitis mice treated with HHL hydrogel. (A) PCA analysis. (B) PCoA analysis. (C) LDA score. Table S1: The DAI scoring rules; Table S2: Primers sequence of ZO-1, Occludin, Claudin-1 and β-actin.
