# Supplementary material for: Fabrication of the Rapid Self-Assembly Hydrogels Loaded with Luteolin: Their Structural Characteristics and Protection Effect on Ulcerative Colitis
Source: Foods. 2024 Apr 4;13(7):1105. doi: 10.3390/foods13071105 (PMC11011723; doi:10.3390/foods13071105)
Supplement: Supplementary file 1 [file foods-13-01105-s001.zip › foods-2913319-supplementary.pdf]

Supporting Information

Table.S1. Disease Activity Index Scoring Criteria

Weight loss	Fecal hardness	Fecal bleeding	Vigor, fur, postural behavior	Score
no weight loss	normal	no obvious bleeding	normal	0
0.1-5%	loose stools	a little blood in the feces	slightly poor condition	1
5-10%	mild diarrhea	blood was often seen in the feces	moderately poor condition	2
≥10%	severe diarrhea	all feces were bloody	Seriously poor condition	3

Table.S2. Specific Primer Sequences for RT-qPCR Analysis

Genes	Forward (5′-3′)	Reverse (5′-3′)
ZO-1	5′-CCAGAGCCTCAGAAACCTCA-3′	5′-GCAGGAAGATGTGCAGAAGG-3′
Occludin	5′-GCGGAAAGAGTTGACAGTCC-3′	5′-TGCCTGAAGTCATCCACACT-3′
Claudin-1	5′-ACGGTCTTTGCACTTTGGTC-3′	5′-GGGAGAGGAGAAGCACAGTT-3′
β-actin	5′-ATCACTATTGGCAACGAGCG-3′	5′-TCAGCAATGCCTGGGTACAT-3′

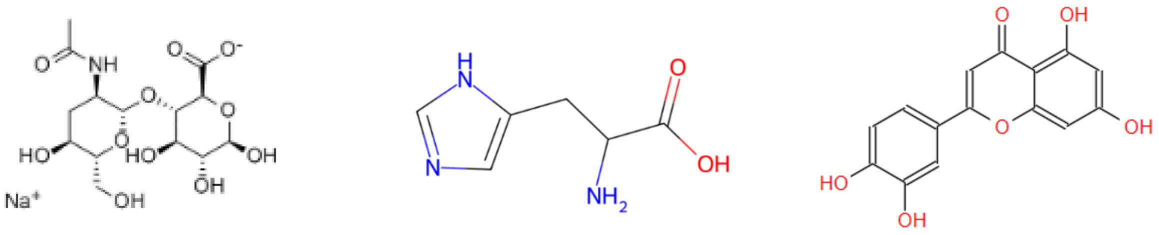

Figure S1. The structural information of HA，His and LUT.

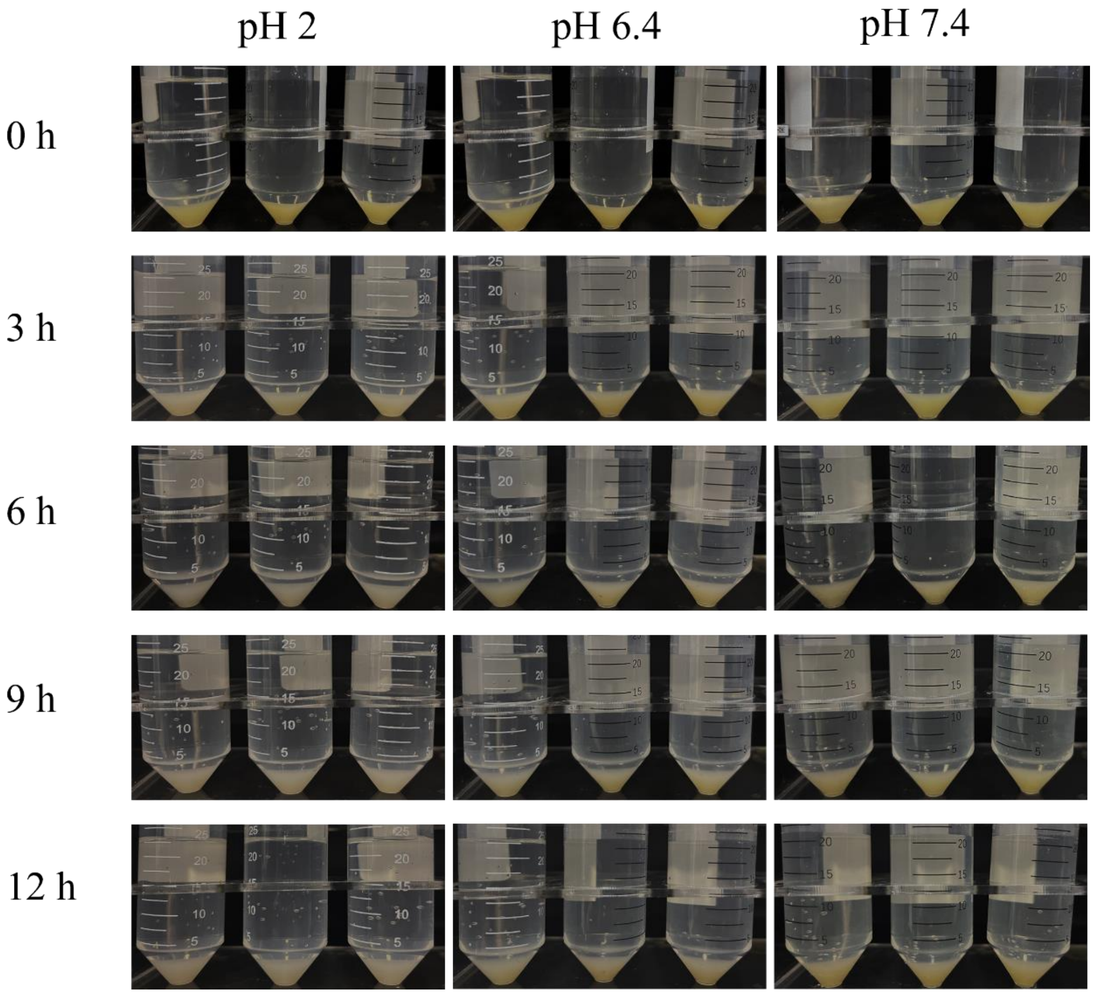

Figure S2. The pH stability of HHL hydrogels.

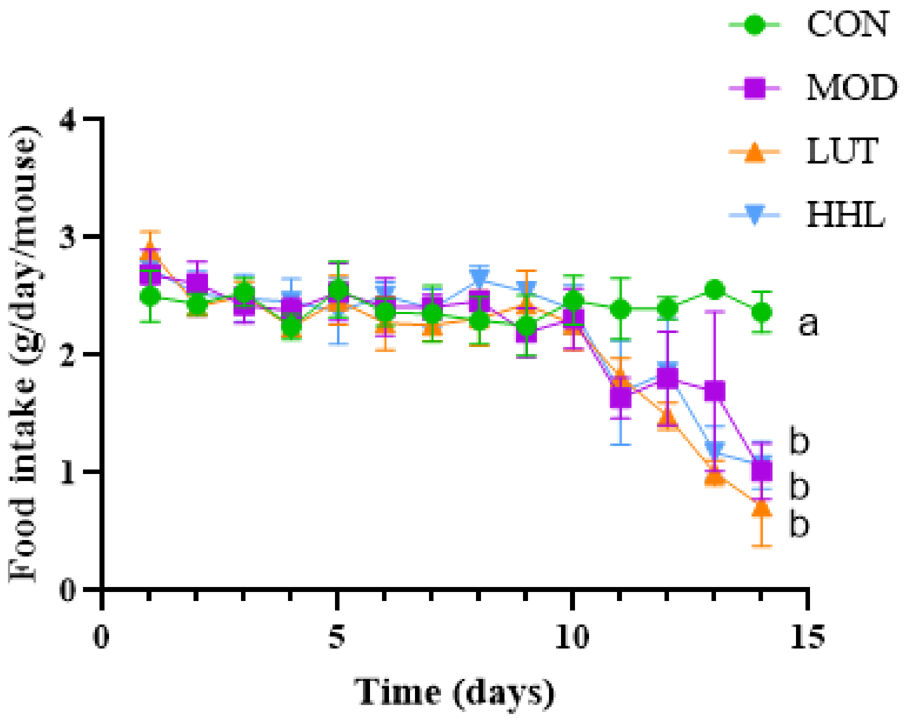

Figure S3. The food intake during animal experiments

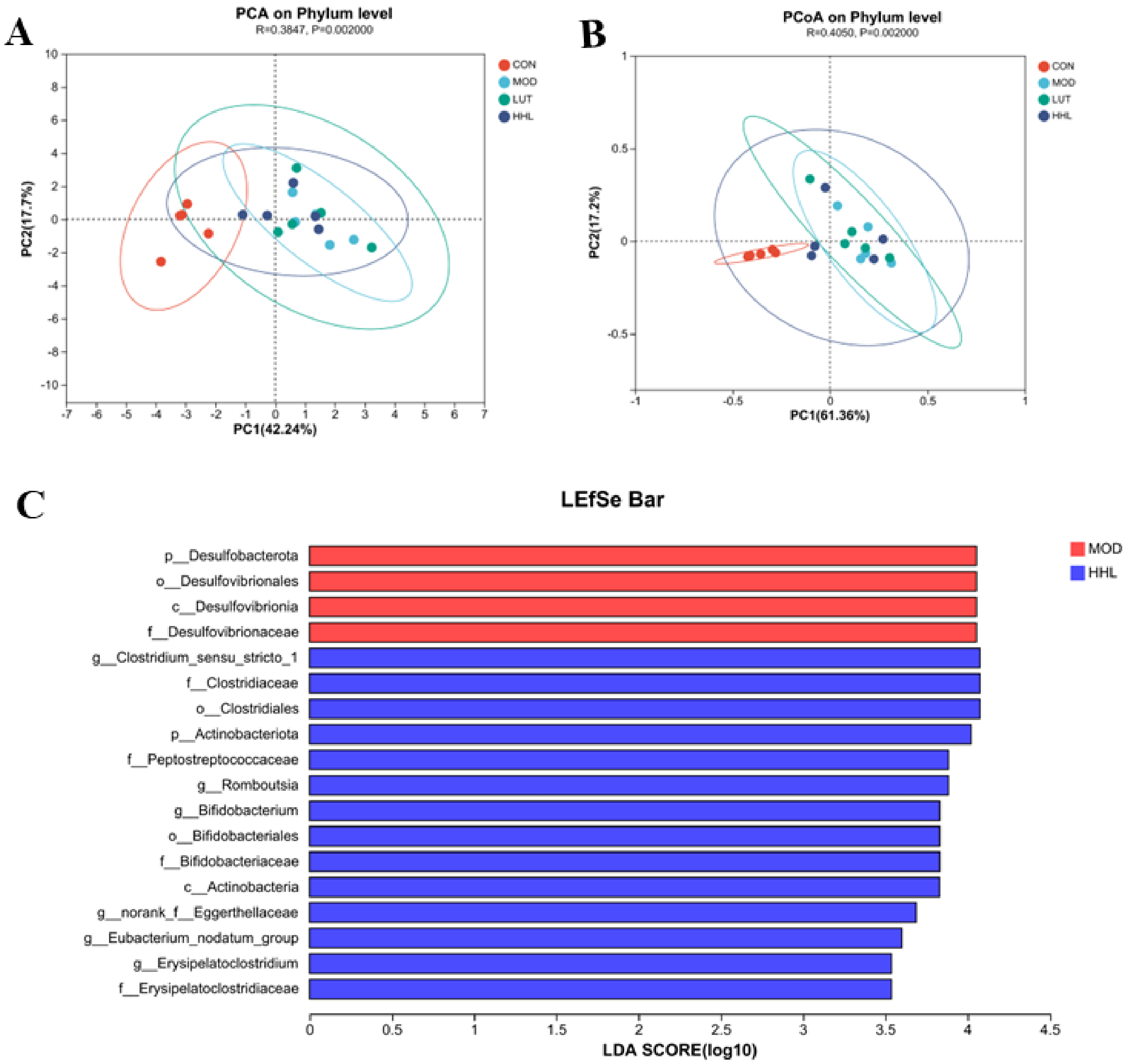

Figure S4. Gut microbiota composition and differential species analysis of DSS induced colitis mice treated with HHL hydrogel. (A) PCA analysis. (B) PCoA analysis. (C) LDA score.
